# Supplementary material for: New calculation model and application research on weak water-flushed zones distribution prediction in radial flow well patterns of heterogeneous oil reservoirs
Source: PLoS One. 2025 May 22;20(5):e0323177. doi: 10.1371/journal.pone.0323177 (PMC12097644; doi:10.1371/journal.pone.0323177)
Supplement: S1 File — Some of the symbols involved in equation derivation may not be easy to understand, so they are explained in supporting file 1. (DOCX) [file pone.0323177.s001.docx]

**Symbol annotation**

Q—quantity of flow, cm^3^/s

$p$—pressure, 10^5^Pa

K—permeability of formation, μm^2^

$\mu_{w}$—viscosity of water phase, mPa·s

$\mu_{o}$—viscosity of oil phase, mPa·s

$R$—seepage resistance, (mPa·s)/(μm^2^·cm)

$r_{e}$—radius between injection and production well, cm

$r_{w}$—radius of production well, cm

$r_{f}$—radius between waterflood front and injection well, cm

$R_{w}$—water phase seepage resistance, (mPa·s)/(μm^2^·cm)

$R_{o}$—oil phase seepage resistance, (mPa·s)/(μm^2^·cm)

$R_{ow}$—total seepage resistance in the two-phase oil-water region, (mPa·s)/(μm^2^·cm)

$\bar{S_{w}}$—average water saturation of formation, %

$S_{wc}$—irreducible water saturation of formation, %

$S_{wf}$—water saturation at waterflood front, %

$\bar{S_{w}^{Lwo}}$—average water saturation of oil-water two-phase region in low-permeability layer, %

$K_{L}$—permeability of low-permeability layer，μm^2^

$K_{H}$—permeability of high-permeability layer，μm^2^

$r_{fL}^{max}$—maximum advance radius of waterflood front in low-permeability layer, cm

$r_{fH}$—advance radius of waterflood front in high-permeability layer，cm

$R_{L}^{min}$—minimum total resistance in the low-permeability layer, (mPa·s)/(μm^2^·cm)

$R_{L}$—seepage resistance in the low-permeability layer, (mPa·s)/(μm2·cm)

$R_{H}$—seepage resistance in the high-permeability layer, (mPa·s)/(μm^2^·cm)

$C_{R}^{i}$—seepage resistance ratio between the low-permeability and high-permeability layers in i_th_ iteration when the waterflood front break through in high-permeability layer, dimensionless factor

$R_{L}'$—the seepage resistance in the low-permeability layer after breakthrough, (mPa·s)/(μm2·cm)

$R_{H}'$—the seepage resistance in the high-permeability layer after breakthrough, (mPa·s)/(μm2·cm)

$C_{R}^{i'}$—the initial seepage resistance ratio between the low-permeability and high-permeability layers after breakthrough, dimensionless factor

$K_{ro}^{\bar{S_{w}}}$—effective permeability of oil phase at formation average water saturation, dimensionless factor

$K_{rw}^{\bar{S_{w}}}$—effective permeability of water phase at formation average water saturation, dimensionless factor

$K_{ro}^{S_{w}}$—effective permeability of oil phase at water saturation of certain section, dimensionless factor

$K_{rw}^{S_{w}}$—effective permeability of water phase at water saturation of certain section, dimensionless factor

$\varphi_{B}$—volume fraction of the weak waterflood zone in the low-permeability layer, dimensionless factor
